# The constellation of skeletal deformities in a family with mixed types of mucopolysaccharidoses: Case report

**DOI:** 10.1097/MD.0000000000004561

**Published:** 2016-08-12

**Authors:** Ali Al Kaissi, Jochen Hofstaetter, Gerlinde Weigel, Franz Grill, Rudolf Ganger, Susanne Gerit Kircher

**Affiliations:** a First Medical Department, Ludwig Boltzmann Institute of Osteology at the Hanusch Hospital of WGKK and AUVA Trauma Centre Meidling, Hanusch Hospital; b Paediatric Department, Orthopaedic Hospital of Speising; c Institute of Medical Chemistry, Medical University of Vienna, Vienna, Austria.

**Keywords:** case report, Maroteaux-Lamy syndrome (MPS VI), radiographs, Sanfilippo syndrome (MPS IIID), skeletal deformities

## Abstract

**Introduction:**

A 13-year-old child was clinically diagnosed with mucopolysaccharidosis type VI—Maroteaux–Lamy syndrome (MPS VI) at the age of 5 years, and the diagnosis was confirmed biochemically and genetically (homozygous mutation in *ARSB* gene). At that time, his older brother manifested with increasing severe mental retardation. His urinary glycosaminoglycan excretion in urine was elevated, but there was only 1 mutation in the *ARSB* gene defining him as a healthy carrier of MPS VI. The 15-year-old boy was born with dysmorphic facial features, cleft lip and palate, and multiple contractures associated with profound skeletal deformities manifested, severe mental retardation, and seizures, leading to the diagnosis of cerebral palsy from birth on.

Clinical and radiographic phenotypic characterization was the baseline tool to document the older sibling, parents, and relatives, all of them examined at the Orthopaedic Hospital of Speising, Vienna, Austria. The family history (from maternal and paternal sides) showed >10 subjects with variable clinical histories of hyperactivity and attention deficit disorder, depression, and a diversity of skeletal abnormalities, such as dysplastic spondylolisthesis, discovertebral degeneration, osteopenia, osteophytosis, and progressive degeneration of the weight bearing zones (mostly developed at middle age).

**Methods:**

Eleven patients in a family with interrelated marriages (two male siblings of 15 and 13-year-old), parents and relatives over three generations were enrolled. One of the siblings was diagnosed with Maroteaux-Lamy syndrome at the age of five-years and mutation of the *ARBS* gene has been encountered. The older sibling manifested at birth craniofacial abnormalities associated with multiple contracture and seizures. Cerebral palsy was the suggested diagnosis. Clinical and radiographic phenotypes were the baseline tool to document the older sibling, parents and relatives at the orthopaedic Hospital of Speising, Vienna, Austria. These were followed by whole Exome sequencing in three family subjects.

**Results:**

A series of genetic studies in the older sibling showed homozygous mutation in *GNS* gene compatible with MPS IIID. Both parents are first related and were found to be heterozygous for *N*-acetylglucosamine-6-sulfatase *GNS* gene. Family history showed more than 10 subjects with variable clinical presentations such as dysplastic spondylolisthesis, disco-vertebral degeneration, osteopenia, osteophytosis, and progressive degeneration of the weight bearing zones (mostly developed at middle age).

**Conclusion:**

Owing to the multiple systemic involvements, a genetic cause was suspected and a molecular genetic investigation by using whole-exome-sequencing method in 3 family subjects (trios) was performed: the 15-year-old boy and his parents. A homozygous splice-site-mutation in the *GNS* gene could be found, compatible with mucopolysaccharidosis–Sanfillipo syndrome (type IIID). Both parents are first related and were now found also to be heterozygous for the *GNS* gene mutation found in their older son. Therefore, both parents are heterozygous carriers for the *ARSB* gene mutation but also the *GNS* gene mutation. In the son with MPS VI, no mutation in the *GNS* gene was found, but the brother with MPS IIID was heterozygous for the *ARSB* gene mutation.

We presume that the intrafamilial variability of clinical signs in different family members could be the result of various mutations in the *ARSB*/*GNS* genes in the carriers or potential modulating effects of other genes or differences in genetic backgrounds.

## 1 Introduction

Mucopolysaccharidoses (MPSs) are rare inherited lysosomal storage disorders caused by enzymatic defects in the catabolism of glycosaminoglycans (GAGs). Presently, there are 11 different enzymatic defects associated with 7 different types of MPS (from I to IX). The lack of enzymatic activity in the degradation of the different types of GAGs leads to tissue-specific intracellular accumulation of substrates, such as heparan sulfate, dermatan sulfate, or keratan sulfate. Clinically, patients present—after an uneventful birth and a short period of normal development—with multisystemic complications associated with organ-specific dysfunction secondary to the intracellular substrate accumulation. ^[1]^

Mucopolysaccharidosis type VI or Maroteaux–Lamy syndrome (Online Mendelian Inheritance in Man no. 253200) is a form of MPS with severe somatic changes, but mental retardation is rare. Onset is around 2 to 3 years with growth retardation, large head, kyphosis, hip dysplasia, genu valgum, and pectus carinatum. Before that growth arrest, there might be a period of accelerated growth with advanced bone age. ^[2]^ Cloudy corneae and coarse facies develop. Radiological features resemble those of Hurler syndrome (mucopolysaccharidosis type I). Hypoplasia of the odontoid process leading to atlanto-axial subluxation has been reported. ^[3]^ The biochemical defect is a deficiency of *N*-acetylgalactosamine-4-sulfatase (arylsulfatase B) leading to increased urinary excretion of dermatan sulfate. The *ARSB* gene on chromosome 5q14.1 is responsible for this type of MPS and homozygous or compound heterozygous disease-causing mutations have been demonstrated. ^[4]^ The clinical spectrum varies from severe to attenuated forms, but a severe subtype with rapid deterioration and severe deformity before the age of 10 years could be observed.

MPS III or Sanfilippo syndromes comprise a group of lysosomal storage disorder caused by an impaired degradation of heparan sulfate. Four subtypes have been defined, each caused by deficiency of a different enzyme: heparan N-sulfatase (type A), alpha-*N*-acetylglucosaminidase (type B), acetyl-coenzyme A, alpha-glucosaminide acetyltransferase (type C), and *N*-acetylglucosamine-6-sulfatase (type D). Compared with the other MPSs, Sanfilippo syndrome is characterized by severe central nervous system degeneration and relatively mild somatic changes. ^[1]^ The first symptoms usually manifest between 2 and 6 years of age, and death typically occurs during the second or third decade of life. For the MPS IIID (Sanfilippo syndrome type III D, Online Mendelian Inheritance in Man no. 252940), Kresse et al and Beesley et al defined the enzymatic defect in the *GNS* gene on chromosome 12q14.3, which encodes for the *N*-acetyl-glucosamine-6-sulfatase.[ ^5^ ^6^]

## 2 Methods

The study protocol was approved by the Medical University of Vienna (Ethics Committee, EK Nr. 921/2009); in addition, informed consent was obtained from the family guardian.

**Figure 1:**
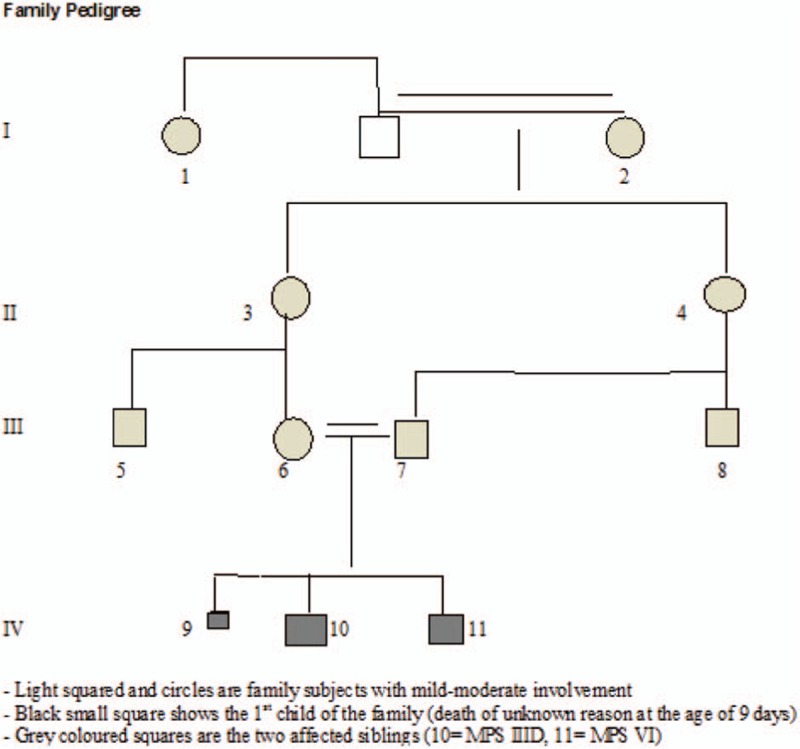
Family pedigree (11 patients over 4 generations, 5 females and 6 males).

Eleven patients over 4 generations (5 females and 6 males) of age range from 0 to 71 years were investigated. Four family subjects (the 2 siblings and parents) were examined via phenotypic characterization, followed by investigation of urinary glycosaminoglycans and genotyping of the older boy and the parents. The rest of the family subjects were evaluated via photographs, conventional radiographs, MRI, and CT scan. Like any other orthopedic hospital, diverse forms of orthopedic problems were the incentive of these families to seek advice. Children, parents, and relatives with frequent hospital admissions were given high priority for a comprehensive studies and phenotypic characterization. The latter is our baseline tool toward the recognition of diverse forms of disabilities in correlation with serious heritable disorders (Fig. 1).

The index patient (please refer to family pedigree) is now a 13-year-old boy born full term by cesarean section (CS) owing to polyhydramnion. Via ultrasound examination at the gestational age of 14 to 15 weeks, spina bifida occulta was recognized. At birth, his weight was 3240 g and his length was 50 cm. At the age of 10 days, urinary tract infection because of reflux was diagnosed and he was operated at the age of 3 months. At the age of 3 years and because of his dysmorphic facial features, skeletal survey showed dysostosis multiplex and dermatan sulfate in urine was identified. Echocardio Doppler showed mitral-valve stenosis. MPS was identified, but the type was not confirmed and no genetic counseling was arranged for the parents. At the age of 4 years, he developed myelopathy because of atlanto-axial instability and occipitocervical fusion was performed accordingly. At the age of 5 years, he was introduced to a specialized metabolic center. DNA testing was performed and a homozygous stop mutation in the *ARSB* gene could be found (exon 5: c.979C > T; p.G327∗). These genetic findings confirmed the diagnosis of MPS VI (Maroteaux-Lamy-syndrome). When his elder brother was diagnosed to have MPS IIID, the index patient also was re-diagnosed and showed no mutation in the *GNS* gene as found in his brother (Table 1).

**Table 1:**
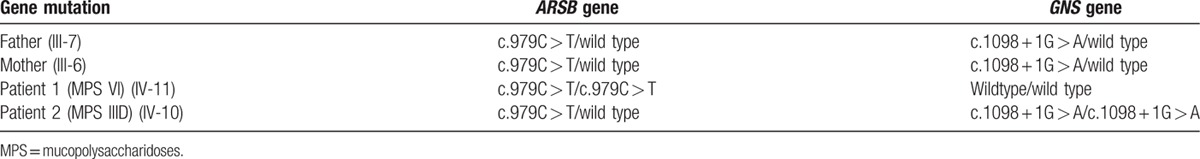
Genetic findings in the siblings and parents for the both MPS-causing genes.

The older brother (subject IV, 10) was born at 35 weeks of gestation via CS, with a birth weight of 2450 g and length 50 cm. At birth, he manifested a constellation of malformation complex of cleft lip and cleft palate, facial dysmorphic features, hypoglycemia, hypacusis, and spastic quadriplegia with convulsions. Anticonvulsive drugs were administered to overcome his fits. Cerebral MRI imaging showed periventricular leucomalacia as result of ischemic encephalopathy. Cerebral palsy was the suggested diagnosis.

At the age of 7 years and at the time of the diagnosis of his younger sibling (IV,11), he also was tested for mucopolysaccaridosis. Urinary glycosaminoglycans were found to be elevated, but no investigations of the different fractions were performed. In accordance to the diagnosis of his brother, molecular genetic testing for MPS VI—Maroteaux–Lamy syndrome by Sanger sequencing method was performed. As he only showed 1 heterozygous mutation in the *ARSB* gene according to his brother, the diagnosis of MPS VI could not be proven, and the diagnosis of spastic cerebral palsy was confirmed.

**Figure 2:**
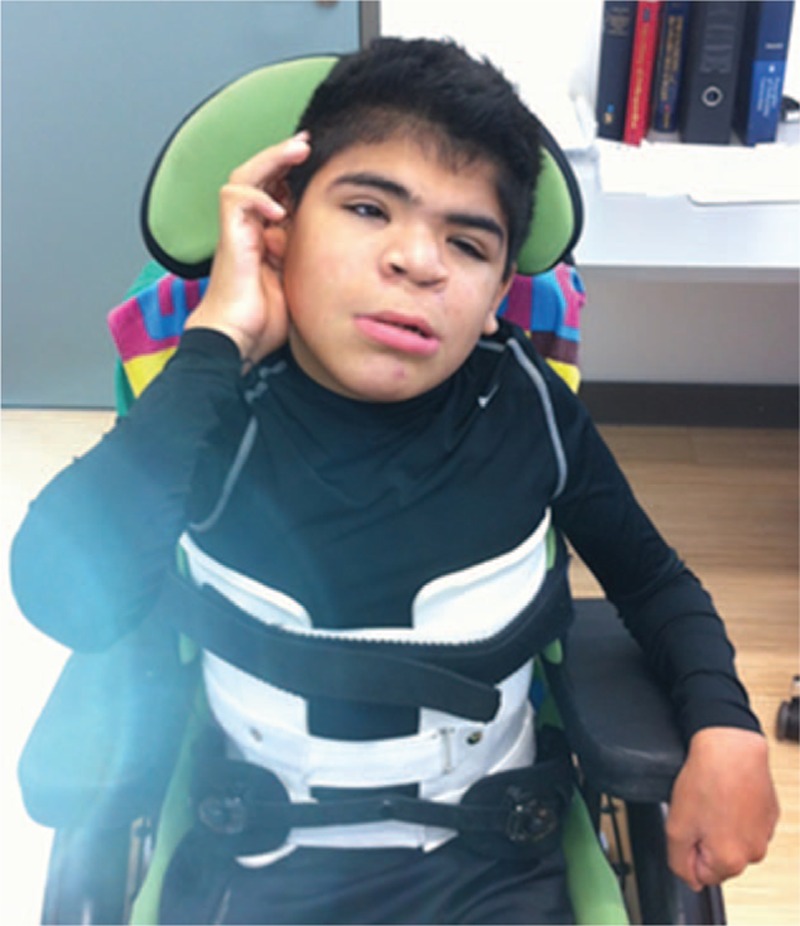
The phenotype of the 15-year-old sibling (IV,10) showed growth deficiency (−4SD) OFC (+2SD), large head in comparison with short stature, thick condensed hair, narrow frontal area, facial asymmetry, hypotelorism, depressed nasal bridge and large flat nose, long philtrum, a repaired cleft lip, a thin upper lip, and large mandible. Severe mental retardation, hearing loss, and bilateral strabismus were associated with multiple contractures.

At the age of 15 years (IV,10), the child was seen for the first time at the Pediatric Department in the Orthopedic Hospital in Speising, Vienna, Austria. He was referred because of severe hip pain. Clinical examination showed severe growth retardation and a relative large head in comparison with short stature (−4SD, OFC was +2SD), evident craniofacial dysmorphic features, thick condensed hair, thick eyebrows, narrowing of temporal area, facial asymmetry, hypotelorism, depressed nasal bridge and large flat nose, cleft lip/palate, long philtrum, wide mouth, thin upper lip, and large mandible associated with multiple contractures and severe mental retardation (Fig. 2).

**Figure 3:**
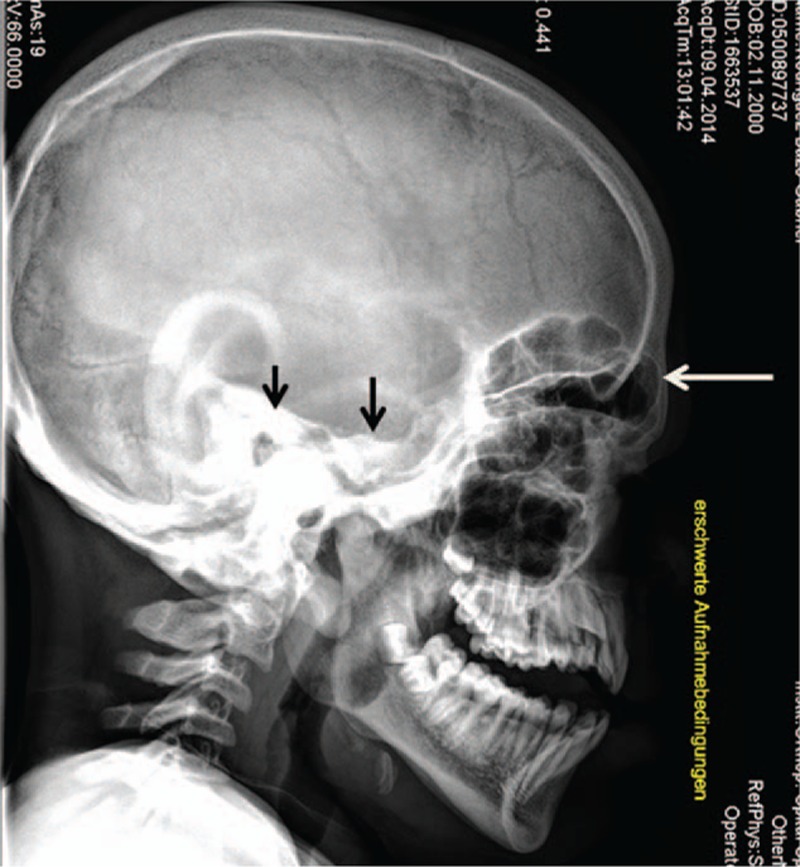
Lateral skull radiograph subject (IV,10) showed thickened sclerosed calvaria, large cella turcica, and hyperostosis of the skull base (arrows).

**Figure 4:**
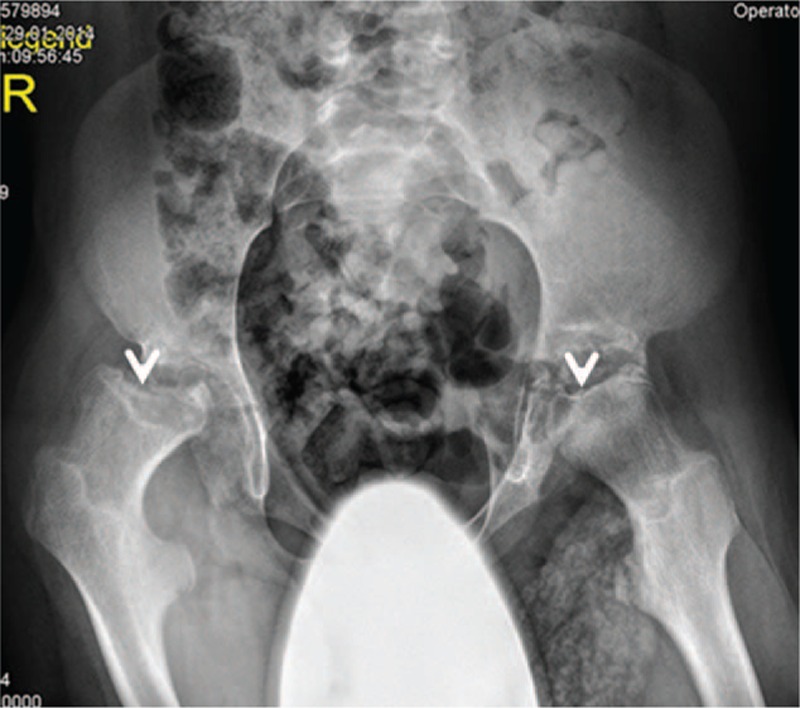
Anterior posterior pelvis radiograph, subject (IV, 10) showed femoral necks in valgus position, hypoplastic iliac bodies, and collapse of the capital femoral epiphyses (arrows) associated with unilateral dislocation of the right hip.

**Figure 5:**
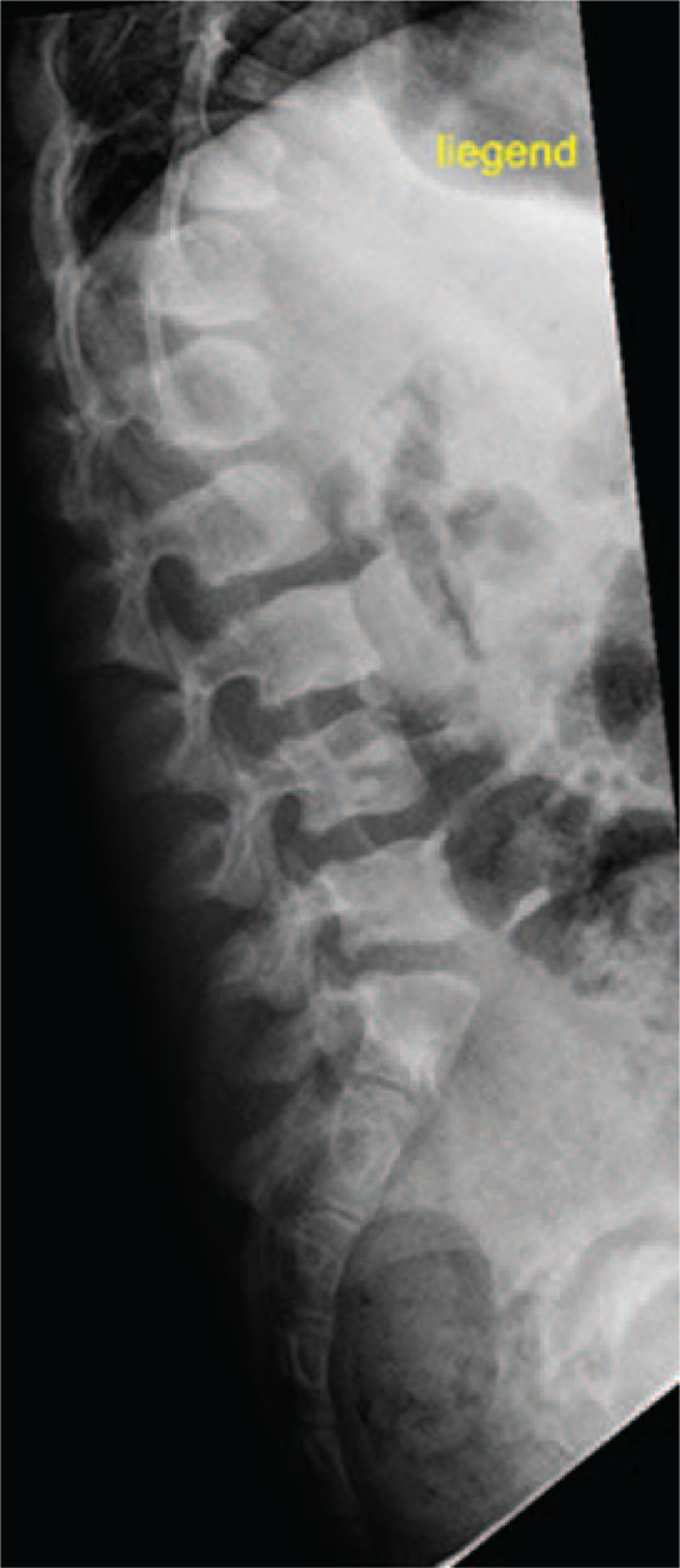
Lateral spine radiograph subject (IV,10) showed anterosuperior hypoplasia and ovoid configurations of T10 to T12. T12 showed marked posterior end plate hypoplasia and L1 to L5 showed anterior end plates concavities.

On the basis of skeletal survey, lateral skull radiograph showed thickened sclerosed calvaria, large sella turcica, and hyperostosis of the skull base (Fig. 3). Anterior posterior (AP) pelvis X-ray showed femoral necks in valgus position, hypoplastic iliac bodies, and collapse of the capital femoral epiphyses (Fig. 4). Lateral spine radiograph showed anterosuperior hypoplasia and ovoid configurations of the vertebrae T10 to T12. T12 showed marked posterior end-plate hypoplasia and L1 to L5 showed anterior end plates concavities (Fig. 5).

## 3 Results

We extensively analyzed the clinical and the radiographic phenotypes of the elder sibling (IV,10), which are typical signs of dysostosis multiplex observed in lysosomal disorders. Owing to the severe mental retardation combined with many changes in organs such as skeleton with signs of dysostosis multiplex, a genetic investigation with whole-exome-sequencing was performed. With this, a homozygous spice-site-mutation between exone 9 and intron 9 in the *GNS* gene was found (c.1098 + 1G > T). This confirmed a MPS IIID and therefore a second type of MPS in this family, MPS IIID (Table 1).

**Figure 6:**
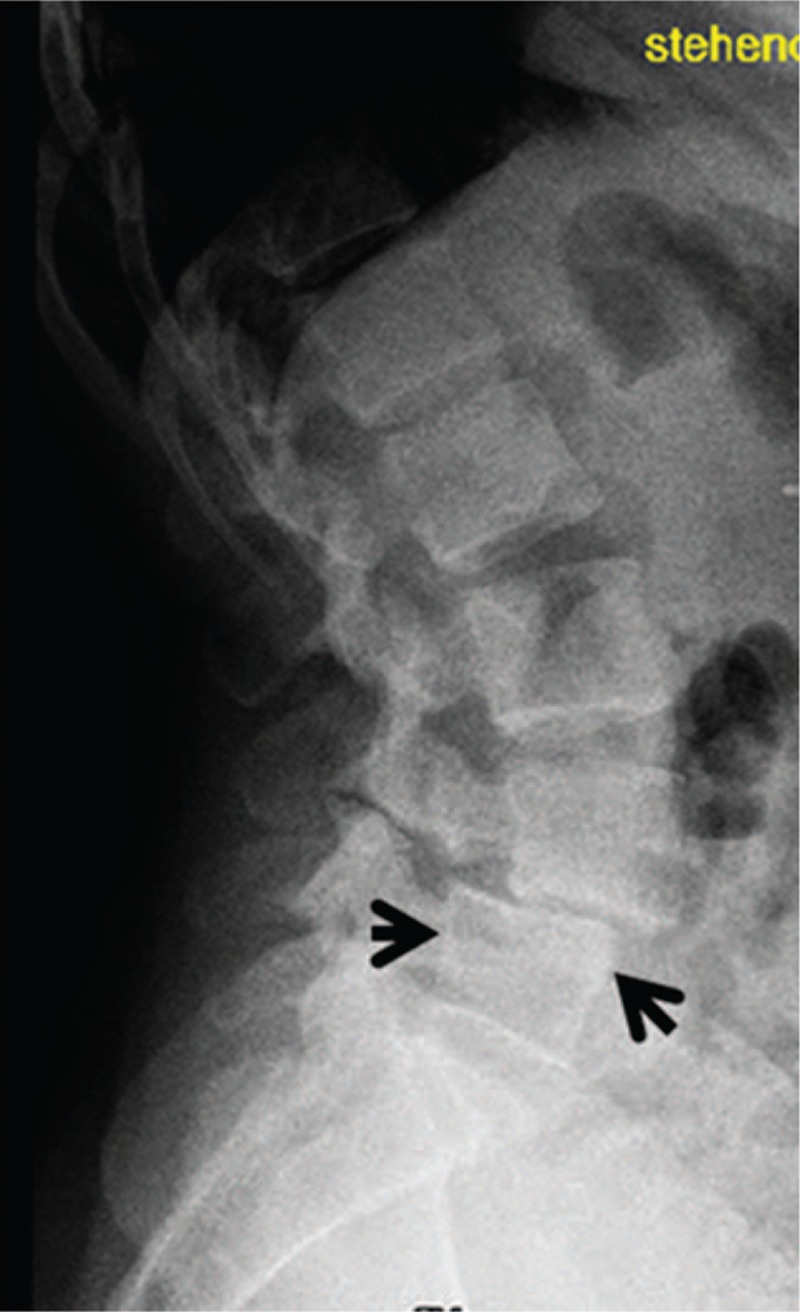
Lateral spine radiograph of the mother (III,6) showed a bilateral break in the pars interarticularis (lucency shown by arrow head). The latter was the reason to develop forward slippage of L5 on the S1; she is a heterozygous carrier of disease causing mutations in the *ARSB* gene as well as in the *GNS* gene, which denote an impaired lysosomal degradation of dermatan sulfate and heparan sulfate in the fully developed diseases MPS VI and MPS IIID.

**Figure 7:**
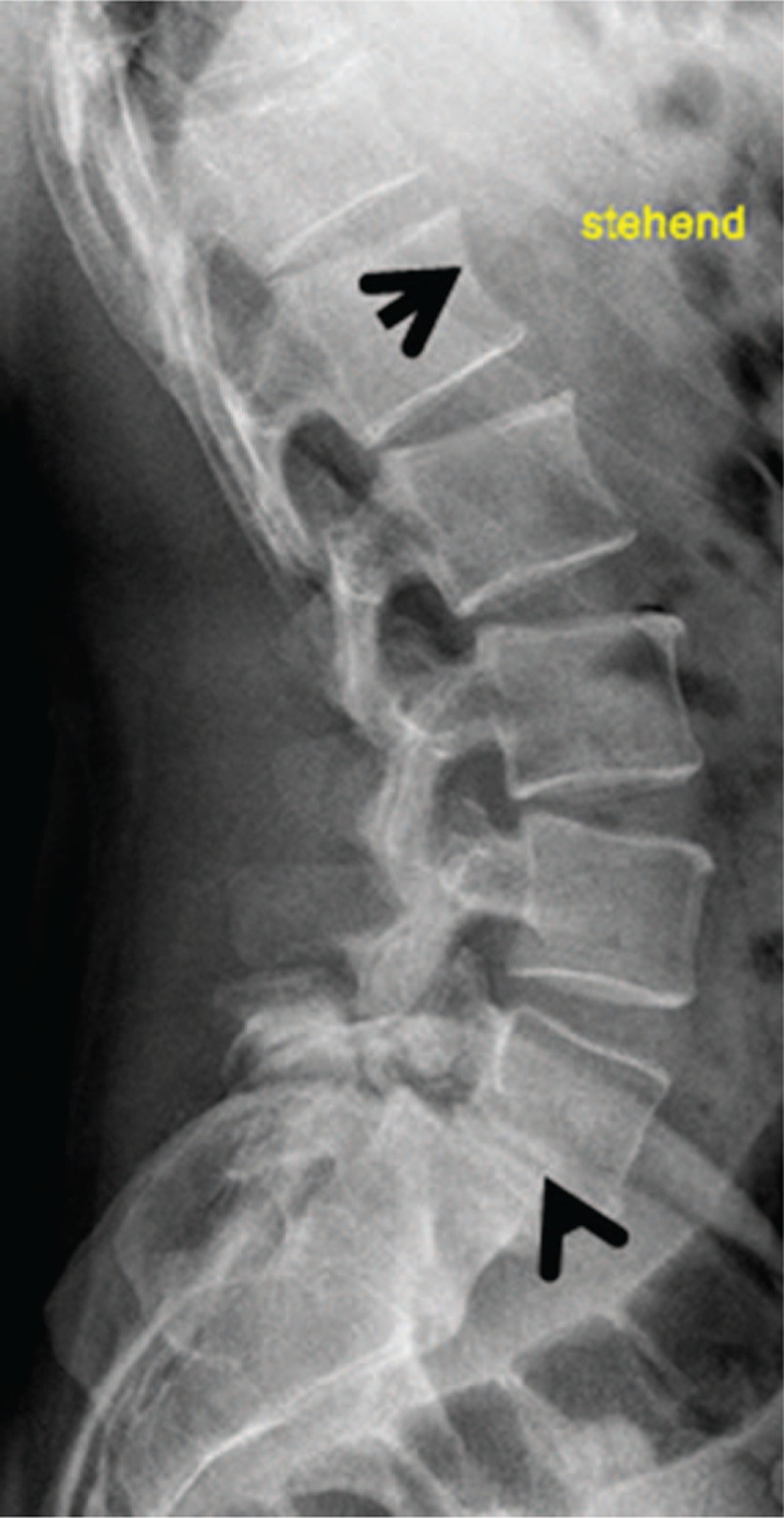
Lateral spine radiograph of the father (III,7) showed notching of the anterior end plates of the lumbar vertebrae associated with grade 2 - spondylolisthesis (forward slippage of L5/S1) because of congenital abnormality of the facets and the pars-interarticularis (arrow). Also the father is a heterozygous carrier of disease-causing mutations in the *ARSB* gene as well as in the *GNS* gene, which denotes an impaired lysosomal degradation of dermatan sulfate and heparan sulfate in the fully developed diseases MPS VI and MPS IIID.

Examination of the parents (subjects III, 6 and 7) showed both manifesting with short stature (-2SD) and mild coarse facies, but with normal intelligence. Both had a history of severe back pain since young adulthood. Lateral lumbar spine radiograph of the mother showed a bilateral break in the pars interarticularis (lucency shown by arrow head). The latter was the reason to develop forward slippage of L5 on the S1 (Fig. 6). AP pelvis radiograph showed osteoarthritis and an increased level of calcification in favor of facet joint arthritis. Lateral spine radiograph of the father (subject III, 7) showed grade 2 SL-vertebral body above subtends 1/2 of the AP diameter of the vertebral body below (Fig. 7).

Both parents showed a normal urinary GAG excretion, and there was no abnormal heparan sulfate or dermatan sulfate fraction seen. After years when carrier status for the *ARSB* gene was identified because of the diagnosis in the first son, both parents now were found also to be heterozygous for the *GNS* mutation of their second son (Table 2).

**Table 2:**
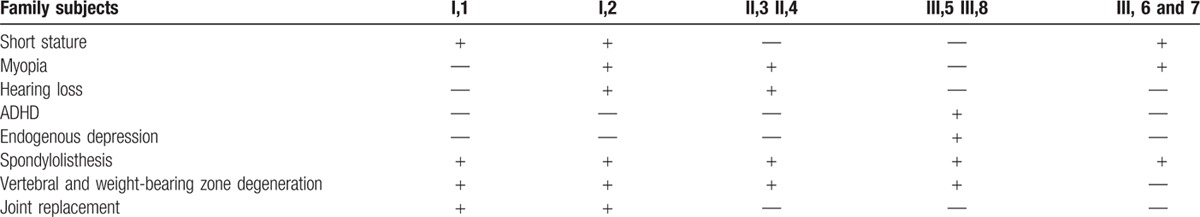
Various skeletal and extraskeletal abnormalities in different family subjects.

## 4 Discussion

The MPSs constitute the largest group of lysosomal storage diseases. The intracellular degradation of complex sugar-containing compounds by lysosomal enzymes is abnormal in this group of diseases, leading to intracellular accumulation of only partly degraded substrates. The MPSs are subdivided based on their enzyme deficiency and the type of substance that accumulates. The GAGs heparan sulfate, dermatan sulfate, and keratan sulfate are the mucopolysaccharides that accumulate and are excreted by the urine. Biochemical analysis of the different fractions in urine can lead to the diagnosis of the specific MPS. Many techniques have been applied to isolate the GAG from the urine. The results of abnormal GAGs vary with the different types of MPS. The excretion typically elevated in MPS VI is dermatan sulfate, and in MPS IIID is heparan sulfate. All types of MPSs show skeletal changes known as dysostosis multiplex and lead to an abnormal short stature. The skull is enlarged, with a thick calvarium, the ribs are oar-shaped and broader anteriorly than posteriorly. The vertebral bodies are hypoplastic and anterosuperior with ovoid configurations. Gibbus, scoliosis, and kyphosis are frequent findings, the iliac wings are flared, and the acetabulae are dysplastic. Coxa valga is common.[ ^1^ ^7^]

Maroteaux-Lamy syndrome (MPS VI) is caused by a deficiency of *N*-acetylgalactosamine-4-sulfatase (arylsulfatase B) leading to increased excretion of dermatan sulfate. The *ARSB* gene has been isolated and mutations demonstrated. ^[4]^ There is abnormal accumulation of the GAG dermatan sulfate. The disease usually manifests at 2 to 3 years of age, when shortness of the trunk and limbs, genu valgum, lumbar kyphosis, and pectus carinatum become apparent. Thorne et al (2001) found that canal stenosis in MPS VI at the level of foramen magnum is more common complication. The biochemical defect is a deficiency of *N*-acetylgalactosamine-4-sulfatase (arylsulfatase B) leading to increased excretion of dermatan sulfate. The gene has been isolated and mutations demonstrated. ^[4]^ About 10% of the world's MPSVI-population (105 patients) were molecular genetically tested by Karageorgos et al, ^[8]^ and mutations were found in 83 patients. Enzyme replacement therapy using recombinant arylsulfatase B has been shown to improve ambulatory ability and joint pain and stiffness, although positive effects on pulmonary and cardial functions are observed. GAG excretion in the urine was decreased by 76%. ^[9]^

Sanfilippo syndrome (MPS III) is a group of 4 autosomal recessive enzyme deficiencies, all leading to an inability to metabolize heparan sulfate. MPS IIIA is caused by a defect in the lysosomal enzyme sulfamidase (Blanch et al, 1997). MPS IIIB is caused by a deficiency of alpha-*N*-acetylglucosaminidase.[ ^10^ ^11^] MPS IIIC results from a deficiency of acetyl-coenzyme A: alpha-glucosaminide-*N*-acetyltransferase. ^[12]^

MPS IIID is caused by a deficiency of N-acetylglucosamine-6-sulfatase. ^[13]^ Mutations in the *GNS* gene result in the lysosomal storage disorder MPS IIID. For all phenotypes, MPS III is a progressive disease with 3 phases that begin after a period of apparently normal development. Sleep problems and disturbances are almost universal associated with extremely irregular sleep pattern on polygraphic recordings.[ ^13^ ^14^] In the first phase, generally starting between the ages of 1 and 3 years, a slowing or plateauing of cognitive development becomes apparent; often speech is more noticeably affected than other cognitive functions. ^[15]^

The second phase starts at approximately 3 to 4 years of age and is characterized by progressive cognitive deterioration and the emergence of behavioral difficulties and sleep disturbances. Behavioral difficulties, including hyperactivity, impulsivity, obstinacy, anxious behaviors, and autistic-like behaviors, worsen over time and can become extreme. ^[16]^

The third stage begins, usually in the teenage years, with the onset of severe dementia and motor function decline. Behavioral problems slowly disappear as patients lose locomotion. Swallowing difficulties and spasticity emerge. Patients eventually regress to a fully bedridden and in a vegetative state; they usually die at the end of the second or beginning of the third decade of life. ^[17]^

Mild cognitive impairment may remain stable into the teenage years or even adulthood before progressing. Behavior problems, similar to those seen in severe patients, do emerge in attenuated patients, but may emerge later or be more manageable in degree. The third stage and death usually occur in the fourth to sixth decade of life, with cases of survival being reported at nearly 70 years of age. ^[18]^ Patients with attenuated disease may easily remain undiagnosed until adulthood, as early diagnosis in this population is particularly challenging. In 1 study of all MPS IIIB patients ever diagnosed in the Netherlands, 33 of 52 patients displayed an attenuated phenotype. ^[19]^

In MPS III, hyperactivity may be marked, with extreme restless behavior, temper tantrums, and crying or laughing fits. Impulsivity may be such that patients may have little to no regard for their own safety. Parents may report the need for constant supervision. One characteristic feature of the behavior is that it does not respond or responds poorly to standard stimulant medications and does not respond to behavior-based interventions. Extreme difficulty in falling asleep and frequent night waking further complicates behavioral problems. ^[20]^ Wijburg et al described the difficulties for the clinicians to distinguish MPSIII behavioral problems from ADHD or autism spectrum disorders. ^[21]^ Sun et al described a combined Hurler and Sanfilippo syndrome in a sibling pair. ^[22]^ They discussed the clinical features and the course of the disease. Neither the radiographic phenotypes of the sibling pair nor details for the parents or other family subjects were included.

## 5 Conclusion

The whole clinical and the radiographic phenotype in our current patient (subject, IV, 10) is not akin to any of the above-mentioned studies. Noteworthy clinical and skeletal abnormalities encountered in our current patient are distinctive: the early life multiple contractures, cleft lip and palate, and the severe axial and appendicular skeletal abnormalities. The constellation of the clinical malformations and the constellation of the skeletal abnormalities were not compatible with any previous patients with MPS IIID.

The family subjects (III, 5 and 8 in Table 2) both manifested a history of ADHD and endogenous depression, associated with vertebral and weight-bearing joint degeneration. The constellation of the clinical and mild radiographic abnormalities might be a link to a type of MPS.

Physicians/geneticists should be aware of missing an unsuspected long-term disorder. Diagnosis should not be established unless adequate knowledge of the etiological understanding is available. Every clinical/ radiographic feature should be potentially analyzed in connection with the patients’ conditions.

This article discusses the basic clinical/radiographic and the genetic phenotypes and the interfamiliar variability of MPS. Neither the constellation of Maroteaux-Lamy syndrome and Sanfilippo D syndrome in a pair of siblings nor the interfamilial involvements have been described in the literature. The parents were not only genetic carriers for MPS IIID, but also for MPS VI. Moreover, they manifested somatic involvement of the vertebral column since their early lives. Similarly, the clinical and the radiographic phenotypes in other family subjects raise the possibility of being genetic carriers for 2 MPS types. We wish to stress on, that Sanfilippo syndrome is characterized by severe CNS degeneration and relatively mild somatic disease. In our current patient (patient 2), there were novel findings such as cleft lip and palate and severe dysostosis multiplex. However, the genetic confirmation in >1 MPS gene involved in a pair of siblings adds to the obscure nature of the disease.
